# Antigen-Based Nano-Immunotherapy Controls Parasite Persistence, Inflammatory and Oxidative Stress, and Cardiac Fibrosis, the Hallmarks of Chronic Chagas Cardiomyopathy, in A Mouse Model of *Trypanosoma cruzi* Infection

**DOI:** 10.3390/vaccines8010096

**Published:** 2020-02-21

**Authors:** Nandadeva Lokugamage, Subhadip Choudhuri, Carolina Davies, Imran Hussain Chowdhury, Nisha Jain Garg

**Affiliations:** 1 Department of Microbiology and Immunology, The University of Texas Medical Branch (UTMB), Galveston, TX 77555-1070, USA; nalokuga@utmb.edu (N.L.); suchoudh@utmb.edu (S.C.); ihchowdh@utmb.edu (I.H.C.); 2 Instituto de Patología Experimental, Universidad Nacional de Salta-CONICET, Salta 4400, Argentina; carolina.davies@exa.unsa.edu.ar; 3 Institute for Human Infections and Immunity, UTMB, Galveston, TX 77555, USA

**Keywords:** *Trypanosoma cruzi*, Chagas disease, immunotherapy, cardiomyopathy, fibrosis, oxidative stress, CD8^+^T cells

## Abstract

Chagas cardiomyopathy is caused by *Trypanosoma cruzi* (*Tc*). We identified two candidate antigens (TcG2 and TcG4) that elicit antibodies and T cell responses in naturally infected diverse hosts. In this study, we cloned *TcG2* and *TcG4* in a nanovector and evaluated whether nano-immunotherapy (referred as nano2/4) offers resistance to chronic Chagas disease. For this, C57BL/6 mice were infected with *Tc* and given nano2/4 at 21 and 42 days post-infection (pi). Non-infected, infected, and infected mice treated with pcDNA3.1 expression plasmid encoding *TcG2/TcG4* (referred as p2/4) were used as controls. All mice responded to *Tc* infection with expansion and functional activation of splenic lymphocytes. Flow cytometry showed that frequency of splenic, poly-functional CD4^+^ and CD8^+^ T cells expressing interferon-γ, perforin, and granzyme B were increased by immunotherapy (*Tc.*nano2/4 > *Tc.*p2/4) and associated with 88%–99.7% decline in cardiac and skeletal (SK) tissue levels of parasite burden (*Tc.*nano2/4 > *Tc.*p2/4) in Chagas mice. Subsequently, *Tc.*nano2/4 mice exhibited a significant decline in peripheral and tissues levels of oxidative stress (e.g., 4-hydroxynonenal, protein carbonyls) and inflammatory infiltrate that otherwise were pronounced in Chagas mice. Further, nano2/4 therapy was effective in controlling the tissue infiltration of pro-fibrotic macrophages and established a balanced environment controlling the expression of collagens, metalloproteinases, and other markers of cardiomyopathy and improving the expression of *Myh7* (encodes β myosin heavy chain) and *Gsk3b* (encodes glycogen synthase kinase 3) required for maintaining cardiac contractility in Chagas heart. We conclude that nano2/4 enhances the systemic T cell immunity that improves the host’s ability to control chronic parasite persistence and Chagas cardiomyopathy.

## 1. Introduction

Chagas cardiomyopathy, caused by *Trypanosoma cruzi* (*T. cruzi or Tc*)*,* is a major health concern in Latin America and it is an emerging disease in the United States, Europe, Japan, and other countries [1]. Most of the infected individuals exhibit no clinical symptoms but remain seropositive for their life [2] and serve as reservoir host for maintaining the domestic cycle of *Tc* transmission. Approximately 30%–40% of the infected individuals slowly develop clinical symptoms that progress from cardiac hypertrophic remodeling (i.e., wall thickening) to dilated cardiomyopathy, ultimately resulting in cardiac arrest and death [3]. Currently available anti-parasite therapies exhibit significant toxicity in infected adults and have shown limited-to-no efficacy in arresting the progression of chronic Chagas heart disease [4]. Thus, new therapies to cure, eliminate, and eradicate *Tc* are needed.

The sequencing and annotation of *T. cruzi* genome [5] has led to identification and testing of antigen-based therapies to elicit protective immune responses against the pathogen. We performed biological screening of several candidate antigens, and selected TcG2 and TcG4 for further development as an anti-parasite immunotherapy. The sequences of *TcG2* (>99%) and *TcG4* (92%–99%) were highly conserved in parasite isolates of five out of six *Tc* lineages, expressed in infective and intracellular stages of the parasite, and recognized by parasite-specific cellular and humoral immune responses in multiple *T*. *cruzi*-infected hosts [6,7,8,9]. To provide a proof-of-concept for the immune protective potential of the selected candidates, we cloned *TcG2* and *TcG4* in pcDNA3.1 eukaryotic expression vector, and showed that immunization of mice and dogs with pcDNA3.*TcG2* and pcDNA3.*TcG4* elicited parasite-specific lytic antibodies, Th1 cytokines, and cytolytic CD8^+^ T cell response that are essential for killing infective and intracellular forms of the parasite [10,11,12,13,14]. Recent studies have tested several other antigenic candidates for their immune efficacy against *T. cruzi* infection. Results of these efforts are encouraging and are summarized in recent reviews [15,16,17,18].

One improvement to increase the immunogenicity of an antigen-based immunotherapy is to optimize the delivery vehicle backbone that will increase antigen expression and manufacturing yield and quality, and will follow regulatory compliance standards [19]. Nano-eukaryotic expression plasmids are designed in accordance with the FDA regulatory guidance regarding composition of DNA vectors for immunotherapy (reviewed in [20]). Specifically, these plasmids consist of minimalized prokaryotic sequence and address regulatory safety issues by utilizing RNA-OUT antibiotic-free approach for selection and amplification. Further, nano-plasmids replace the large 1000 bp pUC replication origin with a novel, 300 bp, R6K-derived mini-origin, utilizing an optimized SV40-CMV-HTLV-1 R chimeric promoter intron to drive improved expression of target genes in mammalian cells, consisting of synthetic eukaryotic mRNA leader and terminators to limit DNA sequence homology with human genome and reduce potential integration in chromosomes, as well as offering a high yield of >0.7 g/L when grown in the HyperGRO fermentation process. Thus, nano-plasmids offer the so-called next generation technology for the delivery and expression of antigen-based immunotherapy.

In this study, we designed nano-plasmids encoding *TcG2* and *TcG4* (referred as nano2/4) and determined whether nano2/4-based therapy promotes protection against chronic Chagas cardiomyopathy. For this, C57BL/6 mice were challenged with *T. cruzi,* and then were treated with two doses of the candidate nano2/4-immunotherapy. Mice treated with pcDNA3.1 encoding *TcG2* and *TcG4* (referred as p2/4) were included in the study to determine if changes in the vector backbone would alter the immunogenic potential of the selected candidates. We examined whether nano2/4 modulated the host T cell immunity to effectively arrest the chronic parasite persistence, and offered protection from chronic inflammation, oxidative stress, and tissue fibrosis that are hallmarks of Chagas cardiomyopathy.

## 2. Materials and Methods

### 2.1. Ethics Statement

All animal experiments were conducted following the National Institutes of Health guidelines for housing and care of laboratory animals and in accordance with protocols approved by the Institutional Animal Care and Use Committee (protocol number 08-05-029) at The University of Texas Medical Branch at Galveston. All experiments were conducted in biosafety level 2 approved laboratory and all personnel received appropriate ABSL2/BSL2 training.

### 2.2. Composition of Immunotherapy

The cDNAs for *TcG2* and *TcG4* (Genbank: AY727915 and AY727917, respectively) were cloned into pcDNA3.1 eukaryotic expression plasmid, and these have been described previously [10]. Recombinant plasmids were transformed into *Escherichia coli* DH5-α competent cells and purified by anion exchange chromatography by using a Qiagen Endo-free maxi prep kit (Qiagen, Chatsworth, CA) [6]. Purified pcDNA3.*TcG2* and pCDNA3.*TcG4* plasmids were used at a concentration of 25 μg each plasmid per dose (referred as p2/4).

Nano plasmids encoding *TcG2* and *TcG4* were constructed by using a CMV promoter-based eukaryotic expression nano plasmid, NTC9385R-MCS (Nature Technology, Lincoln, NE). Briefly, pcDNA3-*TcG2* and pcDNA3-*TcG4* were used as templates, and a traditional PCR was performed with high fidelity Phusion DNA polymerase and *TcG2*- and *TcG4*-specific oligonucleotides incorporating sequences for SalI and BglII restriction enzymes. The amplified *TcG2* (1–660 bp) and *TcG4* (1–276 bp) cDNAs were resolved on 1% agarose gels, purified by using a DNA extraction kit (Qiagen), and ligated into NTC9385R-MCS plasmid at SalI/BglII restriction sites following standard protocol. Then, the ligated products were transformed into DH5α-derived NTC821601 *E. coli* that offered antibiotic-free, sucrose selectable selection to produce recombinant clones. All clones were confirmed by sequencing at the DNA core facility at Nature Technology. Purified NTC9385R-*TcG2* and NTC9385R-*TcG4* plasmids were used at a concentration of 12 μg for each plasmid per dose (referred as nano2/4).

### 2.3. Mice, Challenge Infection, and Treatment with Immunotherapy

C57BL/6 mice (6-week-old females) were obtained from Jackson Laboratory (Bar Harbor, ME). Trypomastigotes of *T*. *cruzi* Sylvio X10/4 strain were maintained and propagated in C2C12 (ATCC CRL-1772) murine muscle cells. Mice (*n* = 5 per group per experiment) were randomly distributed in the following groups: (1) no infection, no treatment; (2) *T. cruzi* only; (3) *Tc* infection followed by two doses of p2/4; and (4) *Tc* infection followed by two doses of nano2/4. Mice in group 2, group 3, and group 4 were challenged by intraperitoneal injection of 10,000 trypomastigotes per mouse. Immune therapies were delivered in 50 µL phosphate-buffered saline (PBS) by intramuscular (im) injection in the hind thigh on day 21 and day 42 post-infection (pi). Mice were euthanized at 120–150 days pi, corresponding to early chronic phase, and blood, serum, plasma, and tissue samples were either used immediately or stored at −20 °C or −80 °C for various experiments. The schematic timeline of infection and treatment is presented in Figure S1A.

### 2.4. Flow Cytometry

Freshly collected splenic samples (*n* = 4–5 mice per group) were processed by standard methods, and single-cell suspensions of splenocytes were incubated with red blood cell lysis buffer and washed with cold 1X PBS. Splenocytes (5 × 10^5^ cells/100 μL) were incubated for 10 min at 4 °C with anti-mouse CD16/CD32 Fc block (BD Biosciences, San Jose, CA), washed twice with ice-cold 1XPBS, and resuspended in 50 µL of flow cytometry staining buffer (00-4222-26, eBioscience, San Diego, CA). Cells were labeled for 30 min at 4 °C in the dark with fluorochrome-conjugated antibodies to surface markers. Cells were washed/fixed with cytofix/cytoperm solution for 20 min, washed with cytoperm wash buffer, and utilized for intracellular staining of effector cytokines for 30 min.

For studying the recall immune responses, *T. cruzi* trypomastigotes (1 × 10^9^ trypomastigotes/mL PBS) were lysed by repeated freeze thaw method, and soluble *Tc* lysate (TcL) was used as antigenic source [10]. Splenocytes from mice were suspended in color-free RPMI-1640 medium (5 × 10^5^ cells/100 μL), and incubated with or without TcL (25 μg/mL) at 37 °C, 5% CO_2_ for 48 h. For the measurement of intracellular markers, brefeldin A (10 μg/mL, BD Biosciences) was added to splenocyte cultures for the final 4 h of incubation to block protein secretion. Cells were then labeled with antibodies for surface markers, fixed and permeabilized (as above), and utilized for intracellular staining of cytokines or specific markers [21]. Fluorescence-conjugated antibodies were purchased from BD Biosciences (APC hamster anti-mouse CD3ε clone 145-2C11, BV510 rat anti-mouse CD4 clone RM4-5, BUV395 rat anti-mouse CD8 clone 53-6.7, BV711 rat anti-mouse interferon γ (IFN-γ) clone XMG1.2, BV650 rat anti-mouse CD62L clone MEL-14, BV786 rat anti-mouse CD44 clone IM7, and BV421 rat anti-mouse CD25 clone 7D4), eBiosciences (PE rat anti-mouse granzyme clone NGZB), and Thermo Fisher Scientific (Waltham, MA; FITC rat anti-mouse perforin clone eBioMAK-D). After labeling with antibodies, cells were suspended in stain buffer, and visualized in duplicate on a LSRII Fortessa Cell Analyzer (BD Biosciences), acquiring 100,000–200,000 events in a live cell gate. Tubes containing unstained cells and cells incubated with isotype-matched IgG, FMO (fluorescence minus one), and live/dead stain were included as controls [13]. We used biological sample controls while placing the gating boundaries. Data were further analyzed using FlowJo software (ver. 10.5.3, Tree-Star, San Carlo, CA) [12,13]. Schematic diagram of flow cytometry analysis of splenic cells is presented in Figure S1B.

### 2.5. Real Time RT-qPCR

Heart and skeletal muscle tissue sections (*n* = 5 mice per group) were homogenized (tissue/buffer ratio, 1:60) in Trizol reagent (Invitrogen, Carlsbad, CA). Splenic cells and tissue homogenates were subjected to chloroform/isopropanol/ethanol method to isolate and precipitate total RNA. Total RNA was treated with RNase-free DNase I (AM2222, Ambion, Austin, TX) to remove the contaminating DNA, and analyzed for quality (OD_260/280_ ratio > 1.8) and quantity (OD_260_ of 1 = 40 µg/mL RNA) by using a NanoDrop ND-1000 spectrophotometer. Purified RNA (1 μg) was reverse transcribed using the iScript cDNA synthesis kit (1708841, Bio-Rad, Hercules, CA) and diluted fivefold with nuclease free ddH_2_O. A real time quantitative PCR was performed on an iCycler thermal cycler in a 20 µL reaction containing 1 µL cDNA, 10 µL SYBR green super-mix (1725121, Bio-Rad), and 500 nM of each of the gene-specific oligonucleotides. The thermal cycling conditions were as follows: denature at 95 °C for 15-s and anneal/amplify at 60 °C for 30 s for 40 cycles. Specific product amplification was confirmed in the melt curve analysis from 63 °C to 95 °C. The PCR baseline subtracted curve fit mode was applied to calculate threshold cycle (*Ct*) values, and (*C_t_*) values of target mRNAs were normalized to mean *Ct* values of *Gapdh* (encodes glyceraldehyde 3 phosphate dehydrogenase) housekeeping gene. The relative change in mRNA level of each target gene was calculated by 2^−ΔCt^ [2 ^(−ΔCt sample)^ / 2 ^(−ΔCt of control)^] method [22]. All oligonucleotides are listed in Table S1.

### 2.6. Parasite Burden

Tissue sections from heart and skeletal muscle (10 mg each, *n* = 5 mice per group) were homogenized and total DNA was purified by using a DNeasy Blood and Tissue kit (69504, Qiagen, Germantown, MD). A real-time qPCR was performed on an iCycler thermal cycler with SYBR Green super-mix (Bio-Rad), 50 ng of total DNA, and oligonucleotides specific for *Tc*18SrDNA sequence and murine *Gapdh* sequence [21]. The threshold cycle (*C_t_*) values for *Tc*18SrDNA were normalized to *Gapdh* reference sequence. The relative parasite burden was calculated by following the 2^−ΔCt^ method as above. Oligonucleotides are listed in Table S1.

### 2.7. Histology

Tissue sections were fixed in 10% buffered formalin, dehydrated in graded ethyl alcohol, cleared in xylene, and embedded in paraffin. Paraffin-embedded 5-micron tissue-sections were stained with hematoxylin and eosin (H&E) or Masson’s trichrome. Heart and skeletal muscle tissue slides were imaged at 20× and 60× magnification by using an Olympus BX-15 microscope (Center Valley, PA) equipped with digital camera and Simple PCI software (v.6.0, Compix, Sewickley, PA). Each microscopic field for H&E-stained tissue sections was scored for inflammatory infiltrate/myocarditis as (0) absent/none; (1) focal or mild with ≤1 foci; (2) moderate with ≥2 inflammatory foci; (3) extensive with generalized coalescing of inflammatory foci or disseminated inflammation; of (4) severe with diffused inflammation, interstitial edema, and loss of tissue integrity [23]. Masson’s trichrome-stained tissue sections were scored for fibrosis/collagen deposition as described [24]. Briefly, all pixels with a blue stain were selected to build a binary image, subsequently calculating the total area occupied by connective tissue. Sections were scored on the basis of percentage of Masson’s trichrome stain-positive area (representing fibrosis-positive area) as (0) < 10%, (1^+^) = 10%–25%, (2^+^) = 25%–50%, (3^+^) = 50%–75%, and (4^+^) > 75%. Further, the intensity of staining was scored as (1) weak, (2^+^) moderate, and (3^+^) strong. Combinative multiplicative fibrosis score was calculated by multiplying the score for the Masson’s trichrome-positive area with the score for staining intensity. Histology data are presented as mean score ± standard deviation (SD), derived from three mice per group, at least two slides per tissue, and nine microscopic fields per slide.

### 2.8. Serum Markers of Oxidative Stress and Inflammation

For measuring the antioxidant capacity, serum samples (1:100 dilution in distilled water, *n* = 5 per group per experiment, duplicate analysis per sample) were analyzed by using the Total Antioxidant Capacity Assay Kit (ab65329, Abcam, Cambridge, MA). The assay is based on antioxidant-based conversion of Cu^2+^ ion into Cu^+^, and detection of reduced Cu^+^ ion chelated with a colorimetric probe at 570 nm. Standard curve was prepared by using Trolox, a water-soluble tocopherol analogue, and antioxidant capacity was quantified as molar Trolox equivalents.

Serum levels of lipid hydroperoxides were examined on the basis of their reaction with ferrous ions and formation of ferric ions, and were detected by using thiocyanate ions with the LPO Assay Kit (ab133085, Abcam). Briefly, serum samples (50 µL, *n* = 5 per group, duplicate observations per sample) were sequentially extracted with equal volumes of chloroform and chloroform/methanol mix (2:1, v/v) to purify lipids. Purified lipids (100 µL volume equivalent) were incubated on ice for 5 min with 0.2 M HCl/3% methanolic solution of ammonium thiocyanate, and the change in absorbance was recorded at 500 nm. The amount of lipid peroxidation was quantified by extrapolation from a standard curve prepared with 0.25–5.0 nM 13-hydroperoxy octadecadienoic acid [25].

Myeloperoxidase (MPO) activity was determined with the *o*-dianisidine method, modified for 96-well plates. Briefly, serum samples (10 μg protein, *n* = 5 per group) were added in duplicate, and reaction was started with 6 mM *o*-dianisidine dihydrochloride (Sigma) in 50 mM citrate buffer pH 5 (Sigma) with 0.03% sodium perborate (substitute of H_2_O_2_). After 5 min incubation at room temperature, the reaction was stopped with 3% sodium azide, and the change in absorbance was measured at 460 nm (ε = 11,300 M^−1^·cm^−1^). Results were expressed as units of MPO/mg protein, whereby 1 unit of MPO was defined as the amount of enzyme degrading 1 nmol H_2_O_2_ per minute at 25 °C [25].

The mouse angiotensin I-converting enzyme (ACE), a key aminopeptidase of the renin-angiotensin system, catalyzes the formation of angiotensin II from angiotensin I. We employed Rab0002 mouse ACE ELISA kit (MilliporeSigma, Burlington, MA) according to the manufacturer’s instructions to quantify the ACE levels in serum samples of all groups of mice (*n* = 5, duplicate observations per sample). The change in absorbance was recorded at 450 nm (standard curve: 0.123–30 ng/mL).

### 2.9. Western Blotting

Heart tissue sections were washed with ice-cold 1X phosphate-buffered saline (PBS), suspended at tissue: buffer ratio of 1: 10 (w/v) in 1X radioimmunoprecipitation assay (RIPA) buffer (9806, Cell Signaling Technology, MA, USA), and homogenized on ice using an Omni tissue homogenizer. Tissues were incubated on ice for 30 min and sonicated three times (20 sec each) by using ultrasonic processor XL. Homogenates were centrifuged at 14,000× *g* at 4 °C for 10 min, and supernatants were stored at −80 °C. Protein concentration was determined by Bio-Rad Protein Assay.

Protein lysates (20 μg each) were electrophoresed on a 10% polyacrylamide gel, and proteins were transferred to PVDF membrane by using a Criterion Trans-Blot System (Bio-Rad). Membranes were blocked with 50 mM Tris and 150 mM NaCl (TBS) containing 0.5% bovine serum albumin (BSA), and were incubated at 4 °C for 18 h with primary antibodies from Abcam (Cambridge, United Kingdom; rabbit anti-4-hydroxinonenal (4-HNE), ab46545, and rabbit anti-mouse GAPDH, ab9485; 1:2000 dilution). Membranes were washed and incubated at room temperature for 30 min with horseradish peroxidase (HRP)-conjugated secondary antibody (4050-05, Southern Biotech, Birmingham, AL, 1:5000–1:10,000 dilution). Color was developed by Pierce ECL Western blot substrate, and images were acquired using an Image Quant LAS4000 system (GE Healthcare, Pittsburgh, MA).

For the detection of protein carbonyls, OxiSelect Protein Carbonyl Immunoblot Kit (STA-308, Cell Biolabs, San Diego, CA) was used. Briefly, protein samples (20 μg each) were transferred to membranes as mentioned above. Membranes were washed for 15 seconds with 100% methanol and incubated for 5 min each in TBS containing 20% methanol, 2N HCl, and 1X dinitrophenyl hydrazine/2N HCl to dinitrophenyl (DNP)-derivatize the protein carbonyls. Membranes were then washed with 2N HCl and 100% methanol, blocked as above, and incubated at room temperature for 2 h with rabbit anti-DNP antibody (1:1000 dilution) and for 30 min with HRP-conjugated secondary antibody (1:5000 dilution). Images were developed and acquired as above. All antibodies were diluted in 5% nonfat dry milk/TBS-0.05% Tween 20. Image J software (NIH, Bethesda, MD) was used to perform densitometry analysis of protein bands of interest and GAPDH (loading control) using tissues from a minimum of four mice per group (duplicate analysis per sample).

### 2.10. Immunohistochemistry

Slides with paraffin-embedded 5 µm heart tissue sections were deparaffinized, suspended in 0.01 M sodium citrate buffer (pH 6.0), and incubated for 10 min in a boiling water bath to unmask the antigens. Slides were washed with PBS and incubated for 10–20 min each with Bloxall blocking solution (Vector Laboratories) and 2.5% normal horse serum to quench endogenous peroxidase activity and block non-specific antibody binding, respectively. Next, tissue sections were incubated for 6–18 h with primary antibodies (rabbit anti-4-HNE, ab46545; mouse anti-8-hydroxy-2′-deoxyguanosine (8-OHdG, ab48508; mouse anti-CD68, ab31630; rabbit anti-TGF-β, ab92486; rabbit anti-MMP9, ab3898; and rabbit anti-galectin-3, ab53082) from Abcam (Cambridge, United Kingdom). Slides were washed in 1X PBS and incubated with ImmPRESS Duet Double Staining Reagent (MP-7714, Vector laboratories) containing HRP-conjugated horse anti-rabbit IgG and alkaline phosphatase (AP)-conjugated horse anti-mouse IgG antibodies. Subsequently, tissue sections were stained with ImmPACT DAB EqV HRP (brown color) and ImmPACT Vector Red AP (magenta color) substrates and fixed in VectaMount AQ Aqueous Mounting Medium (H-5501, Vector Laboratories) [26].

As described for Mason’s Trichrome scoring above, tissue slides were scored to capture the percentages of scanned area that were positive for antigen(s) expression (score range 0–4) and exhibited low-to-high intensity of antigen expression (score range: 1–3), and combinative multiplicative score calculated (*n* = 3 mice per group, 2 tissue sections per mouse, 9 microscopic fields per slide).

### 2.11. Statistical Analysis

The Graph Pad Prism ver.5 software was used for data analysis. Data are expressed as mean value ± standard deviation (SD). Significance was calculated by Student’s *t*-test comparing no infection vs. *Tc* only group (annotated as *) and one-way analysis of variance (ANOVA) with Tukey’s post-hoc test or Kruskal–Wallis H/Dunn’s post-hoc test comparing all infected groups and annotated as ^^^ *Tc* vs. *Tc*.p2/4, ^^^*Tc*.nano2/4 and ^&^*Tc*.p2/4 vs. *Tc*.nano2/4. The *p*-values of <0.05, <0.01, and <0.001 are presented with one, two, and three symbol characters, respectively, in the figures.

## 3. Results

C57BL/6 wild-type mice infected with 10,000 parasites (Sylvio X10) exhibited peak parasitemia during 14–45 dpi and developed early phase of chronic pathology by 120 dpi [12,27]. We employed this well-established experimental model to examine the efficacy of TcG2- and TcG4-based immunotherapy delivered in nano or pcDNA3.1 plasmids in providing protection from *Tc* persistence and Chagas pathology. 

### 3.1. Effects of Nano-Immunotherapy on Functional Activation and Recall Response of CD4^+^T Cells in Chagas Mice

**Figure 1:**
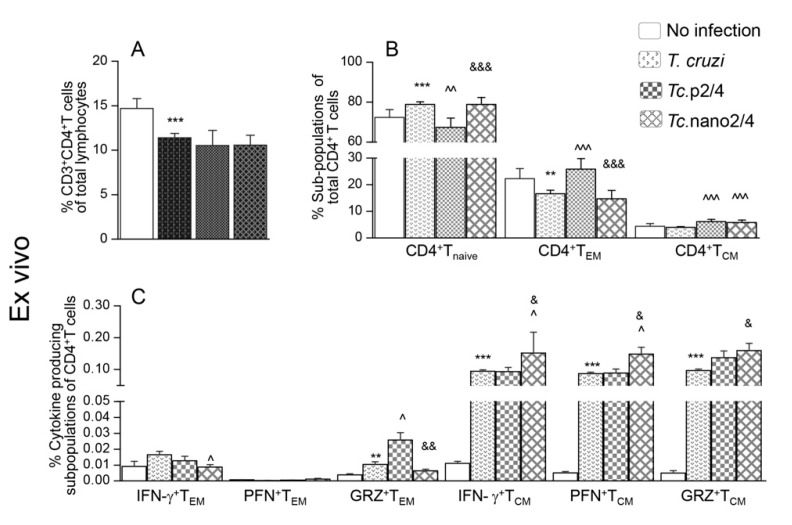

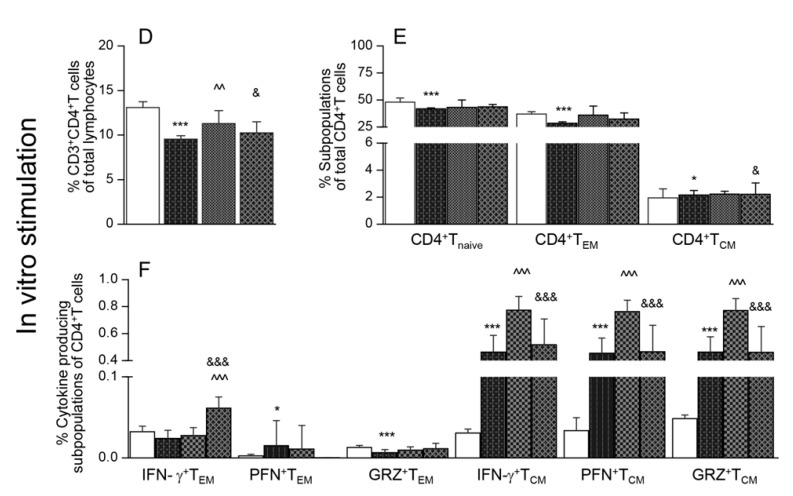
Effect of nano-immunotherapy on ex vivo profile and recall response of splenic CD4^+^ cells in Chagas mice. C57BL/6 mice (6 weeks old) were challenged with *Trypanosoma cruzi* (SylvioX10, 10,000 trypomastigotes per mouse) and treated with two doses of TcG2 and TcG4 cloned in nano-plasmid (referred as nano2/4) at day 21 and day 42 post-infection. Non-infected, infected, and infected mice treated with p2/4 (pcDNA3.1 encoding TcG2 and TcG4) were used as controls. Mice were euthanized at 120–150 days pi (post-infection) corresponding to chronic phase. Freshly collected splenocytes were used for ex vivo profiling (**A**–**C**) or in vitro stimulated for 48 h with *T. cruzi* antigenic lysate (TcL) to evaluate the recall response (**D**–**F**). In all cases, splenocytes were labeled with fluorescent-conjugated antibodies and analyzed by flow cytometry as described in the Materials and Methods section. (**A**,**D**) Shown are splenic percentages of CD4^+^T cells. (**B**,**E**) CD4^+^T cells were analyzed to distinguish naïve (CD44^lo^CD62L^hi^), effector/effector memory (T_EM_, CD44^hi^CD62L^lo^), and central memory (T_CM_, CD44^hi^CD62L^hi^) phenotype. (**C**,**F**) CD4^+^ T_EM_ and T_CM_ subpopulations were analyzed for the expression of IFN-γ, perforin (PFN), and granzyme B (GRZ). Data (mean ± SD) are representative of duplicate observations per sample (*n* = 4–5 mice per group). Significance was calculated by Student’s *t*-test (* no infection vs. *Tc*) and one-way ANOVA/post-hoc tests (annotated as ^^^ *Tc* vs. *Tc*.p2/4, ^^^ *Tc* vs. *Tc*.nano2/4, and ^&^ *Tc*.p2/4 vs. *Tc*.nano2/4). In all bar graphs, the *p*-values of < 0.05, < 0.01, and < 0.001 are presented with one, two, and three symbol characters, respectively.

T cells play an important role in controlling intracellular pathogens such as *T. cruzi*. We therefore first determined if immune therapies modulated the T cell profile in Chagas mice. The overall splenic weight was increased by 33%–53% (range: 0.12-0.14 g) in the infected groups (*Tc* only > *Tc*.nano2/4 > *Tc*.p2/4) as compared to that noted in non-infected controls (average: 0.09 g). The CD3^+^CD4^+^T cells constituted 10.6%–13.9% of the splenic lymphocytes’ population, and these were decreased by 18.0%–24.3% in infected (vs. control) mice (Figure 1A). Except for the *Tc*.p2/4 group in which frequency of CD4^+^T cells of naïve phenotype was decreased and that of effector/effector memory (T_EM_) and central memory (T_CM_) phenotypes were increased, we observed no major changes in the frequencies of these subpopulations in other infected (vs. control) groups (Figure 1B). However, mice in all infected groups (vs. controls) exhibited a significant increase in the functional activation of splenic CD4^+^ T_EM_ and T_CM_ subpopulations. *Tc*.nano2/4 mice exhibited maximal frequency of IFN-γ-, perforin (PFN)-, and granzyme B (GRZ)-expressing CD4^+^T_CM_ subpopulation (^^,&^ *p* < 0.05), and *Tc.*p2/4-treated mice exhibited maximal increase in the frequency of GRZ^+^CD4^+^T_EM_ subpopulation (^^,&^ *p* < 0.05) (Figure 1C).

Upon in vitro stimulation with *Tc* lysate, percentages of CD4^+^T cells did not significantly change in splenocytes of non-infected and infected groups (Figure 1D, compare with Figure 1A). However, we noted a 39.3%–118.8% increase in the frequency of CD4^+^T_EM_ cells (*Tc.*nano2/4 > *Tc* only > *Tc.*p2/4) along with a 35.9%–46.8% and 46.1%–63.7% decline in CD4^+^T_naïve_ and CD4^+^T_CM_ subpopulations, respectively, in infected mice (Figure 1E, compare with Figure 1B). Further, CD4^+^T_EM_ subset of *Tc.*nano2/4 mice was maximally activated for IFN-γ (^^,&^ *p* < 0.001), whereas CD4^+^T_CM_ cells of all infected mice responded to in vitro stimulation with prolific increase in IFN-γ, PFN, and GRZ expression (*Tc*.p2/4 > *Tc*.nano2/4 = *Tc* only; Figure 1F, all *p* < 0.001). In vitro stimulation with TcL did not induce major changes in the responsiveness of splenocytes of non-infected control mice.

Together, the results presented in Figure 1 suggest that *TcG2/TcG4*-based immunotherapy (nano2/4 > p2/4) enhanced the frequency of functionally activated CD4^+^T cells that were capable of responding to parasitic stimulus with increase in IFN-γ and PFN/GRZ production. 

### 3.2. Effects of Nano-Immunotherapy on Functional Activation and Recall Response of CD8^+^ T Cells in Chagas Mice

**Figure 2:**
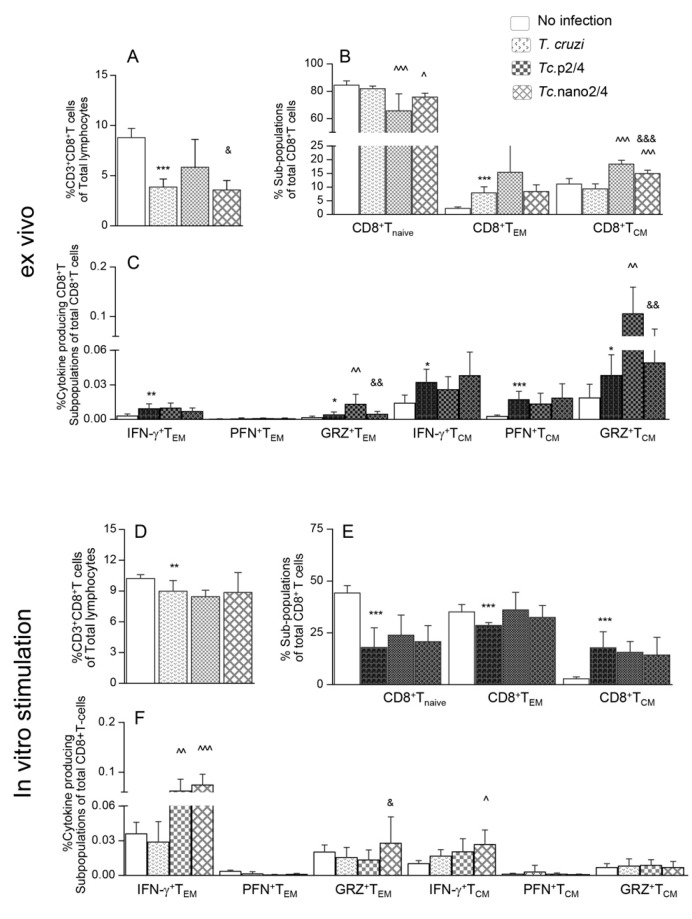
Effect of nano-immunotherapy on ex vivo profile and recall response of splenic CD8^+^ cells in Chagas mice. C57BL/6 mice were challenged with *T. cruzi*, treated with two doses of nano2/4 or p2/4, and euthanized in chronic phase. Splenocytes were used for ex vivo profiling (**A**–**C**) or cultured for 48 h with TcL to evaluate the recall response (**D**–**F**), determined by flow cytometry. (**A**,**D**) Splenic percentages of CD8^+^T cells. (**B**,**E**) Frequency of CD8^+^T cells with naïve, T_EM_, and T_CM_ phenotypes. (**C**,**F**) CD8^+^ T_EM_ and T_CM_ subpopulations were analyzed for the expression of IFN-γ, perforin (PFN), and granzyme B (GRZ). Data (mean ± SD) are representative of duplicate observations per sample (*n* = 4–5 mice per group). Significance was calculated and annotated as described in legends of Figure 1.

Flow cytometry analysis of splenic CD8^+^T cells is presented in Figure 2. These data showed that the splenic frequencies of CD8^+^T cells were decreased by 38.2%–62.1% in infected mice (* *p* < 0.001), and maximal decline was noted in *Tc*.nano2/4 mice (Figure 2A, ^&^ *p* < 0.05). Approximately 60%–80% of the splenic CD8^+^T cells exhibited naïve phenotype in all groups; however, infected/treated mice exhibited 338%–755% and 8.5%–46.4% increase in the frequencies of CD8^+^T_EM_ (*Tc.*p2/4 > *Tc.*nano2/4 > *Tc* only vs. controls) and CD8^+^T_CM_ (*Tc*.p2/4 *> Tc*.nano2/4 vs. *Tc* only and controls, ^ *p* <0.001) sub-populations, respectively (Figure 2B). Further, the frequencies of GRZ^+^CD8^+^T_EM_ and GRZ^+^CD8^+^T_CM_ subsets were higher in *Tc.*p2/4 mice, IFN-γ^+^CD8^+^T_CM_ cells were maximally increased in *Tc*.nano2/4 mice, and PFN^+^CD8^+^T cells were equally increased in all infected groups (Figure 2C).

Upon in vitro stimulation with TcL, splenic CD8^+^T cells of infected (but not of control) mice were increased by 2-3-fold (Figure 2D, compare with Figure 2A). Significant recall response was evidenced by a decline in CD8^+^T_naive_ and an increase in CD8^+^T_EM_ subpopulations in all groups of mice post-antigenic stimulation (Figure 2E, compare with Figure 2B). Yet, infected mice that received immunotherapy exhibited 19.9%–105.9% and 64.3%–161.6% higher frequencies of IFN-γ^+^CD8^+^T_EM_ and IFN-γ^+^CD8^+^T_CM_ subpopulations, respectively, compared to infected/untreated mice (Figure 2F, ^ *p* < 0.01). The frequency of GRZ^+^CD8^+^T_EM_ was increased in *Tc.*nano2/4 mice only (Figure 2F, ^&^ *p* < 0.05).

Together, the results presented in Figure 2 suggest that treatment of infected mice with TcG2- and TcG4-based nano-immunotherapy enhanced the frequency and functional activation of type 1 effector CD8^+^T cells with cytolytic capabilities, and these cells were capable of further expansion in response to secondary antigenic stimulus. Splenic CD8^+^T cells of infected/untreated mice were essentially non-responsive to in vitro stimulation with TcL.

### 3.3. Splenic Expression of Cytokines in Chagas Mice (± Immunotherapy)

To further verify the role of nano2/4 in modulating host response, we analyzed the response of splenocytes of infected (± immunotherapy) and control mice. These results showed a potent increase in the gene expression of *Tnfa* (encodes TNF-α, 6.3-7.6-fold, infected vs. control), *Il1b* (encodes IL-1β, 2.5-12.5-fold, *Tc.*p2/4 > *Tc.*nano2/4 = *Tc* only), *Il6* (encodes IL-6, 2.3-7.6-fold, *Tc.*nano2/4 > *Tc.*p2/4 > *Tc* only), and *Tgfb1* (encodes TGF-β, 2-4-fold, *Tc* only *> Tc.*p2/4 = *Tc.*nano2/4) in splenocytes of infected mice (vs. controls, * *p* < 0.01, Figure S2A–D). Splenocytes of mice given immunotherapy (vs. infected/non-treated) exhibited significantly higher levels of *Il6* (^ *p* < 0.01, Figure S2C) and minimal levels of *Tgfb1* (^ *p* < 0.05, Figure S2D), whereas p2/4 enhanced the splenic *Il1b* expression (^^,&^ *p* < 0.001). These results provide support to the flow cytometry data and show that immunotherapy enhanced the expression of proinflammatory (*Il1b* and *Il6*) cytokines with suppression of profibrotic *Tgfb1* in Chagas mice.

### 3.4. Parasite Persistence and Inflammatory Pathology (± Nano-Immunotherapy)

**Figure 3:**
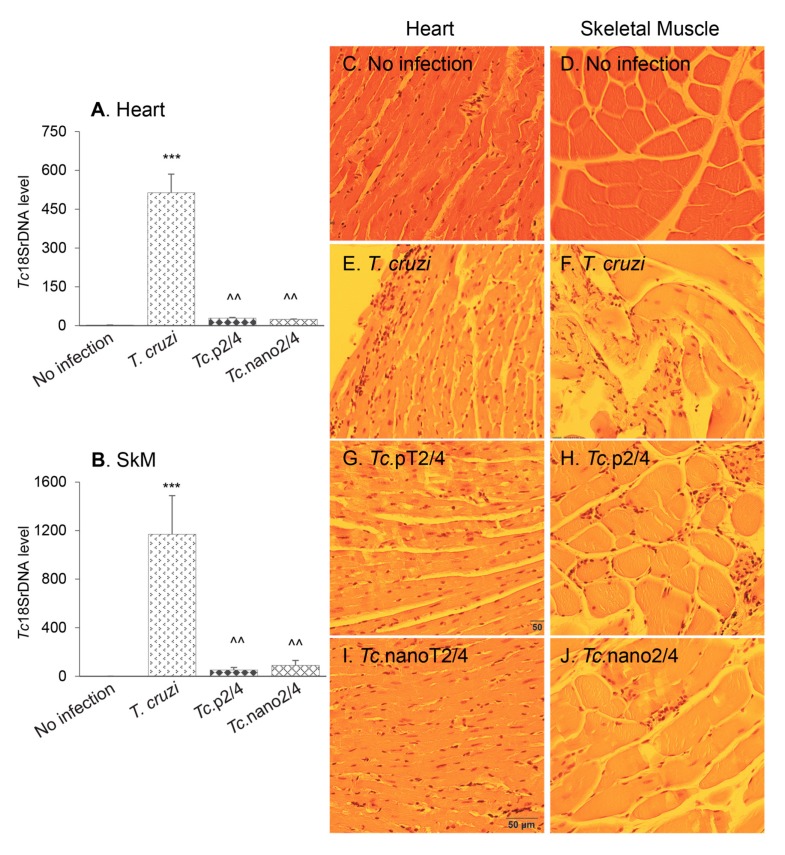
(**A**,**B**) Tissue parasite burden in Chagas mice (± nanotherapy). Mice were challenged with *T. cruzi,* treated with p2/4 or nano2/4, and euthanized as described above. Total DNA was isolated from heart (**A**) and skeletal muscle (**B**) tissue sections and subjected to real-time qPCR amplification of Tc*18SrDNA* sequence (normalized to murine *Gapdh*). Data (mean ± SD) are representative of duplicate observations per sample (*n* = 5 mice per group per experiment). Significance was calculated and annotated as described in legends of Figure 1. (**C**–**J**) Myocardial inflammatory infiltrate in Chagas mice (± nano2/4). Paraffin-embedded 5 µm tissue sections were examined by hematoxylin/eosin staining. Shown are representative images of heart and skeletal muscle tissues from non-infected (**C**,**D**), *T. cruzi-*infected (**E**,**F**), *Tc.*p2/4 (**G**,**H**), and *Tc.*nano2/4 (**I**,**J**) groups of mice (magnification: 20×). Inflammatory score was derived from image analysis of *n* = 3 mice per group, two tissue sections per mouse, and > 9 microscopic fields per section, and presented in Table 1A.

Next, we determined how the changes in systemic T cell immunity affected the parasite persistence and associated inflammatory pathology in heart and skeletal tissues of Chagas mice. Quantitative real-time qPCR evaluation of tissue parasite burden showed that nano2/4 performed better than p2/4 in controlling the cardiac tissue parasite burden (94.3%–95.2% decline) and equally as p2/4 with 92.4%–95.5% control of skeletal muscle levels of parasite burden in chronically infected mice (Figure 3A,B, ^ *p* < 0.01). 

Histology studies showed extensive infiltration of inflammatory cells constituted of macrophages, neutrophils, and T lymphocytes in the heart (average histology score: 2.17, * *p* < 0.001) and skeletal muscle (average histology score: 2.91, * *p* < 0.001) of infected/non-treated mice (Figure 3E,F, Table 1A). Despite an increase in systemic immune response, *Tc.*nano2/4 and *Tc.*p2/4 mice exhibited noteworthy control of inflammatory infiltrate in the heart tissue (average histology score: 0.83-0.91, ^ *p* < 0.01) and skeletal muscle (average histology score: 0.91–1.0, ^ *p* < 0.01) (Figure 3G–J). No significant differences in the overall levels of tissue inflammation were observed between *Tc.*nano2/4 and *Tc.*p2/4 mice, though mice given p2/4 exhibited slightly better preservation of cardiac tissue integrity, whereas nano2/4 was more effective in preserving the integrity of skeletal muscle in infected mice. Parasite DNA and inflammatory infiltrates were not detectable in uninfected control mice (Figure 3A–D). Together, the results presented in Figure 3 and Table 1A suggest that TcG2- and TcG4-based immune therapies were highly effective in reducing the tissue parasite persistence and associated pathological inflammatory infiltrate that are the hallmarks of chronic Chagas disease.

### 3.5. Efficacy of Nano2/4 in Arresting Peripheral and Myocardial Oxidative Stress in Chagas Mice

**Figure 4:**
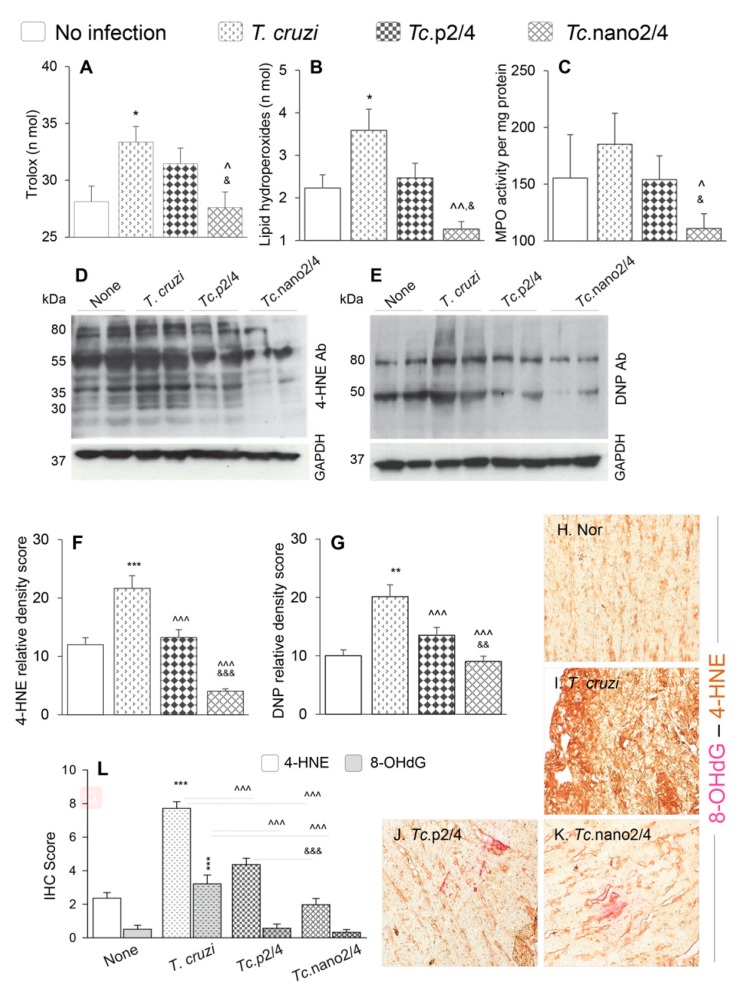
Control of systemic antioxidant and oxidant status in Chagas mice treated with nano-immunotherapy. Mice were infected, treated, and euthanized as described in Figure S1. (**A**–**C**) Shown are serum levels of (**A**) total antioxidant capacity, (**B**) lipid hydroperoxides levels, and (**C**) myeloperoxidase (MPO) activity. Data are presented as mean value ± SD (*n* = 5 mice per group, duplicate observations per sample per experiment). (**D**–**G**) Heart homogenates (20 µg) were resolved by SDS-PAGE. Representative Western blot images for 4-hydroxynonenal (4-HNE) (D) and dinitrophenyl (DNP) derivatized protein carbonyls (**E**) are shown. GAPDH was probed as a loading control. Relative densitometry score for 4-HNE (**F**) and DNP-carbonyls (**G**) was normalized to GAPDH and presented as mean value ± SD (*n* = 4 mice per group, duplicate Western blot analysis per sample). (**H**–**L**) Myocardial tissue sections from different groups of mice were subjected to immunohistochemistry staining. Shown are representative images for 4-HNE/8-OHdG (lipid peroxidation/oxidative DNA damage) levels in cardiac tissue of non-infected (**H**), infected (**I**), *Tc*.p2/4 (**J**), and *Tc*.nano2/4 (**K**) mice at 60× magnification. Bar graph (**L**) shows the immunohistochemistry staining score (mean value ± SD) for 4-HNE and 8-OHdG (*n* = 3 mice per group, 2 tissue sections per mouse, 9 microscopic fields per tissue section, analyzed at 20× magnification). For data presented in bar graphs, significance was calculated and annotated as described in Figure 1.

Reactive oxygen species produced by immune cells and dysfunctional mitochondria in non-immune cells are shown to contribute to pathological oxidative stress in Chagas disease [28]. We therefore determined if immune therapies modulated the peripheral and cardiac levels of oxidative damage in Chagas mice. Serum level of Trolox (marker of antioxidant capacity) was increased by 17.8% but it was not sufficient in arresting the oxidative/inflammatory stress evidenced by 43% and 20% increases in the lipid hydroperoxides content (oxidative stress marker) and myeloperoxidase activity (marker of neutrophil activation), respectively, in Chagas (vs. control) mice (* *p* < 0.05, Figure 4A–C). Treatment with p2/4 resulted in 5.6%, 30.6%, and 16.8%, but non-significant, decline in the Trolox, LPO, and MPO levels, respectively, in infected mice (Figure 4A–C). However, nano2/4 was highly effective in normalizing the peripheral antioxidant capacity to the control levels and oxidative/inflammatory stress below the control levels in Chagas mice (all, ^^,&^ *p* < 0.05, Figure 4A–C).

Myocardial oxidative stress was examined by semi-quantitative assessment of 4-HNE and protein carbonyl adducts by Western blotting. These data revealed the formation of 4-HNE adducts on many proteins of different sizes (range: 30–80 kDa), whereas carbonyl adducts were predominantly detected on two protein bands (50 kDa and 80 kDa) in chronically infected mice (Figure 4D,E). Relative peak density of 4-HNE (35 kDa) and carbonyl (80 kDa) adducts were increased by 80%–100% in infected (vs. control, * *p* < 0.001) mice, and decreased by 39%–81% and 33%–55%, respectively, in infected/treated mice (Figure 4F,G, ^ *p* < 0.001). The nano2/4 was more effective than p2/4 evidenced by elimination of 4-HNE adducts in the myocardium of *Tc*.nano2/4 mice (Figure 4F,G, & *p* < 0.001).

Immunohistochemical staining of tissues was performed to validate the efficacies of immune therapies in controlling the 4-HNE and 8-OHdG, which are critical markers of endogenous oxidative stress and DNA damage, respectively [29]. These data showed extensive increase in 4-HNE (2.5-fold) and 8-OHdG (>5-fold) deposition in myocardium of Chagas mice (Figure 4H,I,L, * *p* < 0.001). Similar to biochemical findings in serum samples and Western analysis of myocardial tissues, immunohistochemical studies also showed that treatment with immune therapies resulted in 45%–75% and 80%–90% control of 4-HNE and 8-OHdG adducts, respectively, in Chagas myocardium (Figure 4J,K,L, all ^ *p* < 0.001), and that nano2/4 was significantly more effective than p2/4 in arresting the cardiac levels of 4-HNE adducts in Chagas mice (Figure 4J,K, ^&^ *p* < 0.001).

Together, the results presented in Figure 4 suggest that nano2/4 efficiently controlled the peripheral and cardiac levels of lipids, proteins, and DNA oxidative adducts that otherwise were pronounced in Chagas mice.

### 3.6. Tissue Fibrosis and Profibrotic Macrophages in Chagas Disease (± Nano Therapy)

**Figure 5:**
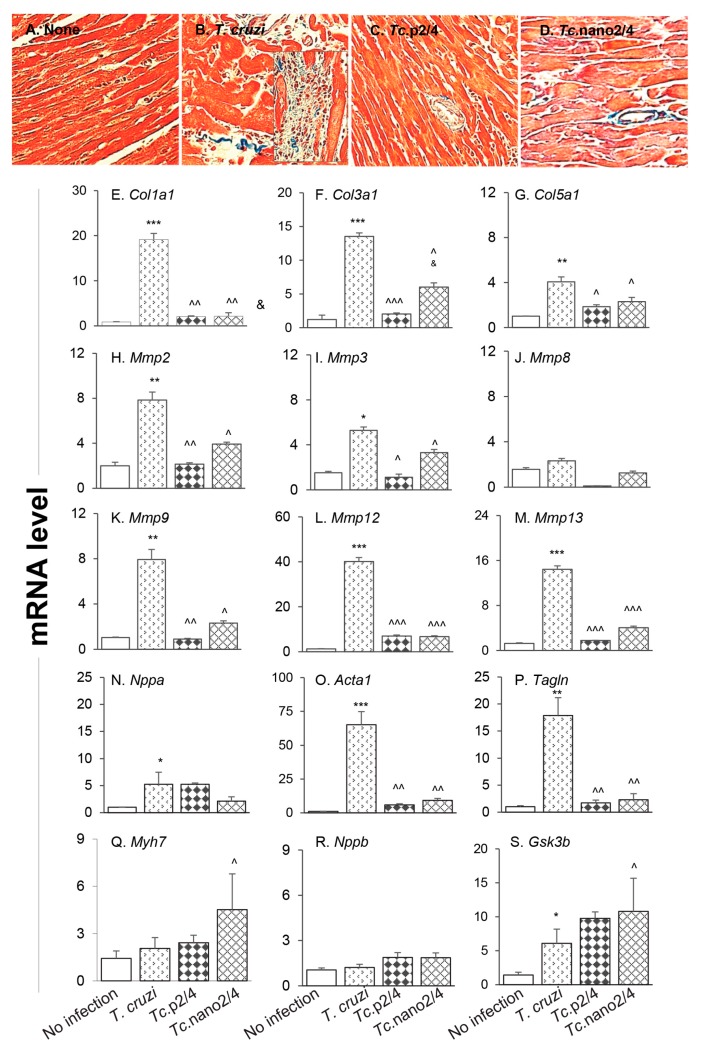
Hypertrophy and fibrosis in Chagas mice (± nano-immunotherapy). Mice were infected, treated, and euthanized as described in Figure S1. (**A**–**D**) Representative images of Masson’s trichrome staining of heart tissue sections from non-infected (**A**), infected (**B**), *Tc*.p2/4 (**C**), and *Tc.*nano2/4 (**D**) groups of mice are shown (magnification: 20×). Fibrosis scores were calculated from *n* = 3 mice per group, 2–3 tissue sections per mouse, and at least 9 microscopic fields per section, and are presented in Table 1B. (**E–S**) Heart tissues were subjected to RT-qPCR evaluation of mRNA levels for collagens (**E**–**G**), metalloproteinases (**H**–**M**), and hypertrophy markers and repair proteins (**N**–**S**). Gene expression data were normalized to *Gapdh* and values are presented as mean value ± SD (derived from *n* = 5 mice per group, duplicate observations per sample). Significance was calculated and annotated as described in Figure 1.

The splenic expression of *Tgfb1* and systemic angiotensin II levels, the major inducers of fibrosis/hypertrophy, were increased by threefold (Figure S2D) and 39% (data not shown), respectively, in Chagas mice, and controlled after treatment with immunotherapy. We therefore set out to determine if cardiac fibrosis, a hallmark of Chagas disease, is decreased in mice treated with nano-immunotherapy. Histological staining with Masson’s trichrome showed intense increase in myocardial infiltration of myofibroblasts and collagen deposition in heart and skeletal muscles of Chagas (vs. control) mice (Figure 5A,B; Figure S3A,B,E; Table 1B; all * *p* < 0.001). RTqPCR studies corroborated the histological findings and showed a significant increase in the expression of pro-fibrotic collagens (*Col1a1, Col3a1, and Col5a1)* in the myocardium (4–18-fold increase, all, * *p* < 0.05, Figure 5E–G) and skeletal muscle (10–34-fold increase, all * *p* < 0.05, Figure S3F–H) of Chagas mice. The mRNA levels for metalloproteinases (*Mmp2, Mmp3, Mmp9, Mmp12, and Mmp13*, Figure 5H-M, 4–40-fold increase, all * *p* < 0.05) as well as the markers of hypertrophic growth, i.e., *Nppa*, *Acta1* and *Tagln* encoding for atrial natriuretic peptide, skeletal muscle α-actin and smooth muscle protein 22α (also called transgelin), respectively, were significantly increased (5–65-fold increase, all * *p* < 0.05, Figure 5N–P) in Chagas myocardium. No significant changes in the mRNA levels for *Mmp8, Myh7* (encodes myosin heavy chain β, contractile protein), and *Nppb* (encodes brain natriuretic peptide) were observed (Figure 5J,Q,R), whereas the expression of protective *Gsk-3b* (encodes glycogen synthase kinase 3 β) was increased by two-fold in Chagas heart (* *p* < 0.05, Figure 5S). In comparison, Chagas mice treated with immune therapies exhibited low-to-no infiltration of myofibroblasts and 7.5–13-fold decline in the myocardial (Figure 5C,D) and skeletal muscle (Figure S3C–E) levels of collagen deposition (all ^ *p* < 0.001, Table 1B). Further, treatment with *TcG2/TcG4*-based immune therapies resulted in 7–14-fold decline in the expression of genes encoding for collagens (Figure 5E–G, Figure S3F-H), metalloproteinases (Figure 5H–M), and hypertrophy markers (Figure 5N–P) in Chagas mice (all ^ *p* < 0.05). In general, nano2/4 was more effective in controlling the fibrotic/hypertrophic responses that was associated with a significant increase in the protective *Myh7 and Gsk-3b* expression levels (Figure 5Q,S, ^ *p* < 0.05). 

**Figure 6:**
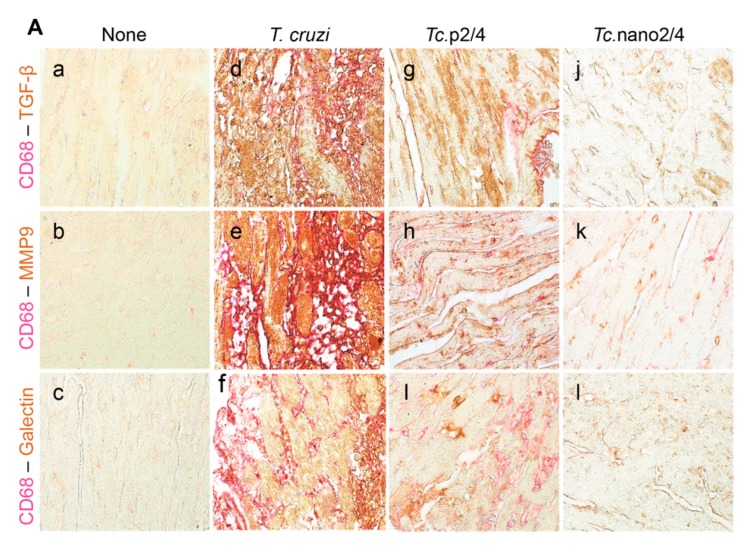

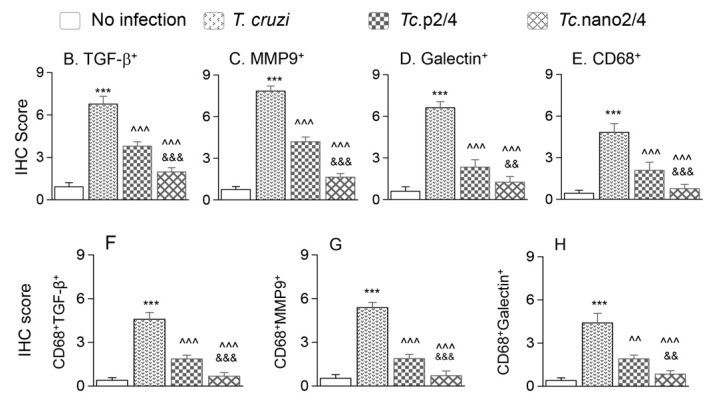
Nano2/4-immunotherapy controlled tissue fibrosis through modulation of macrophages in Chagas disease. Mice were infected, treated, and euthanized in chronic phase. Paraffin-embedded 5 µm heart tissue sections were subjected to immunohistochemical (IHC) staining. (**A**) Shown are representative images of heart tissue from non-infected (panels (a–c)), *T. cruzi*-infected (panels (d–f)), *Tc.* p2/4 (panels (g–i)), and *Tc.*nano2/4 (panels (j–l)) groups of mice (60× magnification). Tissues were stained for CD68 (macrophage marker) along with TGF-β (a,d,g,j), MMP9 (b,e,h,k), and galectin-3 (c,f,i,l). (**B**–**H**) Semi-quantitative IHC scores for TGF-β (**B**), MMP9 (**C**), galectin-3 (**D**), and CD68 (**E**) are presented. Co-localization of CD68 with TGF-β (**F**), MMP9 (**G**) and galectin-3 (**H**) was scored as described in the Materials and Methods section. IHC score values are presented as mean ± SD (*n* = 3 mice per group, 2 tissue sections per mouse, 9 microscopic fields per tissue section, analyzed at 20× magnification). Significance was calculated and annotated as described in Figure 1.

We performed immunohistochemical staining to verify the effect of nano2/4 in controlling tissue fibrosis in Chagas mice. Myocardial tissue of Chagas mice exhibited extensive distribution and 5–8- fold increase in TGF-β, MMP9, and galectin-3 (myofibroblast marker) levels compared to that noted in non-infected controls (Figure 6A–F, all * *p* < 0.001). Treatment with *TcG2/TcG4*-based immune therapies led to 50%–70%, 50%–80%, and 50–75% control of *Tc*-induced increase in the myocardial levels of TGF-β, MMP9, and galectin-3, respectively (all ^ *p* < 0.001, Figure 6A–D) and maximal positive effects were observed when mice were treated with nano2/4 (^&^ *p* < 0.01).

Because myocardial expression of *Mmp12* and *Mmp13* that are encoded by macrophages was maximally increased (Figure 5L,M), we also performed co-localization studies to examine whether macrophages contribute to profibrotic response in Chagas disease. These results showed that myocardial distribution of CD68^+^ (a macrophage marker) cells was increased by 8–10-fold in Chagas mice (* *p* < 0.001), and controlled by 50% and 80% in *Tc.*p2/4 and *Tc.*nan2/4 mice, respectively (^ *p* < 0.001, Figure 6A,E). Importantly, combinative scoring for co-localization showed that CD68^+^TGF-β+ and CD68^+^MMP9^+^ subpopulations were increased by 8-fold and 10-fold in Chagas myocardium (vs. controls, * *p* < 0.001), and controlled by 55%–80% and 65%–85%, respectively, when infected mice were treated with immune therapies (^ *p* < 0.001, Figure 6A,F,G). Co-localization of CD68 and galectin was also decreased by 55%–75% in infected mice treated with immune therapies (*p* < 0.01, Figure 6H). In all cases, nano2/4 was better than p2/4 in arresting the myocardial infiltration of TGF-β+, MMP9^+^, and galectin-3^+^ CD68^+^ cells of macrophage lineage (all ^&^ *p* < 0.01). Together, these results corroborated the findings presented in Figure 5 and suggested that myocardial infiltration of macrophages producing TGF-β and MMP9, at least partially, contribute to signaling of fibrotic/hypertrophic responses in Chagas myocardium, and that these pro-fibrotic macrophages were controlled in mice treated with immunotherapies (nano2/4 > p2/4).

## 4. Discussion

Immune therapies employ the biological response modifiers to stimulate, restore, or suppress the immune system to fight infection, cancer, and autoimmune diseases, or to protect from side effects of a particular treatment. Potential immune therapies against Chagas disease are envisioned to modulate the immune system to target the infective trypomastigote form of the parasite to prevent or limit the persistence of infection and the intracellular replicative amastigotes to arrest its propagation and re-entry into the blood. Further, an immunotherapy is expected to target all lineages and circulating isolates of the parasite in order to be potentially useful. We found that TcG2 and TcG4, the target antigens used in immunotherapy in this study, are expressed in all mammalian stages of *Tc* [6] and consist of epitopes recognized by antibodies and T cells in mice, dogs, and humans (reviewed in [18]). Remarkably, TcG2 and TcG4 were preserved in five of the six *Tc* lineages (80%–96% homology), affording us confidence that TcG2/TcG4-based treatment can offer protection against diverse *Tc* isolates in the USA and Latin America. In this study, we demonstrated that TcG2/TcG4-based immunotherapy inhibited the chronic persistence of *T. cruzi* and associated inflammation, oxidative stress, and fibrosis in the Chagas myocardium.

Though many investigators including us have shown excellent prophylactic efficacy of subunit vaccines in controlling subsequent infection and associated pathologies, very few studies have tested the concept of immunotherapy for control of Chagas disease (reviewed in [18]). Some studies have tested trypomastigote surface antigen 1 (TSA-1), trans-sialidase (TS), and amastigote surface protein (ASP-2) pertaining to the *trans*-sialidase family as well as Tc52 (glutathione *S*- transferases) and Tc24 (a Ca^2+^ binding protein) for therapeutic efficacy in infected mice. When injected immediately after infection or within 2 weeks post-infection, *Tc52-, TSA-1-*, and *Tc24-*based DNA therapies decreased the parasitemia and mortality from infection in mice [30]. The protection was associated with increase of CD8^+^ T cell proliferation and IFN-γ production [31,32]. However, these same candidates did not control cardiomyopathy in chronically infected mice or dogs (reviewed in [18,33]). DNA therapy encoding ASP2 and TS (individually or in combination) offered no protection against parasite burden, mortality, and cardiomyopathy in infected mice [15,30], despite their known efficacy as a prophylactic vaccine. Others have observed an increase in myocarditis in infected mice inoculated with TSA1 DNA therapeutic vaccine [31]. These studies did not extend to determine why only selective candidates offered a degree of therapeutic efficacy against the parasite, why the therapeutic treatment failed to arrest myocarditis and fibrosis, and how the immune system was geared (or not geared) towards protection.

In our experience, parasite persistence and associated oxidative stress and inflammatory infiltrate into the heart are major pathological events that contribute to cardiac fibrosis and cardiomyopathy development in Chagas disease. The delivery of TcG2/TcG4-based nano-immunotherapy was timed in such a manner that the adaptive immune system was activated against acute parasitemia, but persistence of parasite, inflammation, and oxidative stress did not cause the irreversible tissue damage in the heart of the infected mice. The nano therapy elicited a potent increase in the splenic Th1 cytokines, and functionally activated CD4^+^ and cytotoxic CD8^+^T cells capable of responding to parasite antigenic stimulation with further increase in IFN-γ and PFN/GRZ production. Thus, nano-immunotherapy activated the systemic T cell immunity to control circulating parasites and prevent tissue invasion and tissue injury caused by parasite persistence and the resultant inflammatory infiltrate. Importantly, timing of treatment was critical to target all three hallmarks of Chagas disease and obtaining the successful outcome.

Oxidative stress during Chagas disease could potentially be ameliorated with antioxidants delivered in conjunction with an immune therapy. Treatment with the anti-parasitic drug benznidazole in early-to-late phase of chronic infection is shown to control parasite persistence; however, it did not avert cardiac remodeling and deterioration of left ventricular function in humans and experimental models of Chagas disease [34,35,36]. Adjuvanting the benznidazole with antioxidants, for instance, PBN (phenyl-alpha-tert-butylnitrone), vitamin A, or by genetic overexpression of MnSOD (manganese superoxide dismutase) or GPx (glutathione peroxidase) has been shown to restrain oxidative insult along with parasite burden, and to improve cardiac function [37,38,39]. Likewise, treatment with sildenafil, an inhibitor of phosphodiesterase 5, provided cardioprotection through the preservation of cGMP-PKG activity and antioxidant–oxidant balance in chronically infected mice [40]. Sirtuin 1 (SIRT1) maintains mitochondrial metabolic homeostasis and prevents the over-activation of inflammation [41]. We found that SIRT1 activity was decreased in Chagas mice, and treatment with SIRT1 agonist (SRT1720) or inhibition of PARP1 (poly (adenosine diphosphate-ribose) polymerase 1) that competes with SIRT1 for substrates reduced oxidative and inflammatory stress in infected cells and mice [42]. Moreover, we noted increased efficacy of a therapeutic vaccine in infected mice overexpressing glutathione peroxidase than was observed in infected/wild type mice under similar conditions [39]. Thus, it is possible that nano-immunotherapy designed to achieve a rapid, short-term stimulation of type 1 cellular immunity to attack the persistent parasites, if complemented with antioxidants (e.g., PBN or resveratrol), would offer better efficacy in preserving the tissue homeostasis in Chagas disease.

Myocardial fibrosis is recognized as a strong, independent predictor of adverse outcomes in Chagas patients [43]. Cardiac fibrosis occurs with differentiation of cardiac fibroblasts (resident and infiltrating) to myofibroblasts and imbalance of extracellular matrix (i.e., collagens) production and degradation (by metalloproteinases). Hypertrophic growth of cardiomyocytes is routinely associated with increase in the expression of ANP/BNP and a decline in GSK-3β (inhibits hypertrophic growth) and β-MHC (essential for contractile function) [44]. Heart tissue of chronically infected Chagas mice exhibited histological signature of cardiac fibrosis and hypertrophy in this study. Further, MMPs, galectins, and TGF-*β,* noted to be major tissue remodeling players in human Chagas disease [45], were up-regulated in expression in chronically infected mice in this study. Specifically, MMPs (e.g., MMP2, MMP9) that promote tissue levels of profibrotic mediators and epithelial-to-mesenchymal transition, as well as abnormal epithelial cell migration and other aberrant repair processes, were activated in Chagas mice. Remarkably, MMP12 and MMP13 that are primarily produced by macrophages were maximally activated in Chagas myocardium. MMP12 is recognized as degrading soluble and insoluble elastin; inducing pro-osteogenic calcification in aortic valve disease [46]; and playing a role in aneurysm formation, lung dysfunction, and chronic obstructive pulmonary disease [47]. MMP12 deficiency attenuated the angiotensin II-induced vascular injury, tissue infiltration of M2 macrophages, and heart fibrosis in mice [48]. MMP-13 has received attention because of its central role in the cartilage degradation network and it is also proposed to have anti-fibrotic activities in murine models of atherosclerosis [49]. Our studies provided the first clue to the potential role of infiltrating macrophages in signaling cardiac fibrosis in Chagas disease. TGF-β, which signals through both canonical and noncanonical pathways, was attenuated by nano-immunotherapy; however, further studies are needed to examine the exact signaling mechanism that was targeted by nano-immunotherapy in controlling cardiac fibrosis in Chagas disease. 

## 5. Conclusions 

We showed that nano2/4 enhances the systemic T cell immunity to control chronic parasite persistence and Chagas cardiomyopathy. Our findings suggest that immunotherapies, alone or in conjunction with benznidazole or other anti-trypanocidal chemical drugs, offer an opportunity to arrest the chronic progression of symptomatic Chagas disease.

## Figures and Tables

**Table 1:** Semi-quantitative scoring of tissue pathology in Chagas mice (± immunotherapy).

Immunotherapy	Heart Mean ± SD (Score Range)	Skeletal Muscle Mean ± SD (Score Range)
**A. Inflammation score**		
No infection	0	0
*T. cruzi* only	2.17 ± 0.17^***^ (0–3)	2.91 ± 0.08^***^ (1–3)
*Tc*.p2/4	0.83 ± 0.11^^^^^ (0–1)	1 ± 0^^^^^ (1)
*Tc*.nano2/4	0.91 ± 0.08^^^^^ (0–1)	0.91 ± 0.08^^^^^ (0–1)
**B. Fibrosis score**		
No infection	0	0
*T. cruzi* only	5.1 ± 0.48^***^	6.4 ± 0.8^***^
*Tc*.p2/4	0.73 ± 0.02^^^^^	0.74 ± 0.6^^^^^
*Tc*.nano2/4	0.4 ± 0.04^^^^, &^	0.51 ± 0.05^^^^, &^

C57BL/6 mice were challenged with *T. cruzi* (SylvioX10), treated with two doses of TcG2 and TcG4 cloned in nano plasmid (referred as nano2/4), and euthanized at 150 days post-infection. Non-infected, infected, and infected mice treated with p2/4 (pcDNA3.1 encoding TcG2 and TcG4) were used as controls. Histological evaluation of inflammation and fibrosis was performed as described in the Materials and Methods section. Data (mean ± SD) are representative of *n* = 3 mice per group, 2 sections per tissue per mouse, and 9 microscopic fields per section. Significance was calculated by Student’s *t*-test (* no infection vs. *Tc*) and one-way ANOVA/post-hoc test (annotated as ^^^ *Tc* vs. *Tc*.p2/4, ^^^ *Tc* vs. *Tc*.nano2/4 and ^&^ *Tc*.p2/4 vs. *Tc*.nano2/4). The *p*-values of < 0.05, < 0.01, and < 0.001 are presented with one, two, and three symbol characters, respectively.

## Supplementary Materials

The following are available online at https://www.mdpi.com/2076-393X/8/1/96/s1, Figure S1: Schematic of experimental design and Schematic showing gating strategy for CD4^+^ and CD8^+^ T cell phenotypes, Figure S2: Splenic expression of cytokines in Chagas mice (± immune therapy), Figure S3: Skeletal muscle fibrosis in chronically infected mice (± nano immune therapy), Table S1: Oligonucleotides used in this study.
