# Supplementary material for: Antigen-Based Nano-Immunotherapy Controls Parasite Persistence, Inflammatory and Oxidative Stress, and Cardiac Fibrosis, the Hallmarks of Chronic Chagas Cardiomyopathy, in A Mouse Model of Trypanosoma cruzi Infection
Source: Vaccines (Basel). 2020 Feb 21;8(1):96. doi: 10.3390/vaccines8010096 (PMC7157635; doi:10.3390/vaccines8010096)
Supplement: Supplementary file 1 [file vaccines-08-00096-s001.zip › Supp file 1 Table S1.docx]

| Supplementary file 1  **Table 1.** Oligonucleotides used in this study. | | | | | | |
| --- | --- | --- | --- | --- | --- | --- |
| **Gene** | **Protein** | **Accession #** | **Primers** | **Sequence 5'-3'** | **Size (bp)** |  |
| **Amplification of cDNA for cloning in nano vector** | | | | | |  |
| *TcG2* | *T. cruzi* G2 | AY727915 | F | GGAAGGGTCGACGCCACCATGTCGCTTTCATTTATCGAGTCAGGGT | 660 |  |
|  |  |  | R | GGAAGCAGATCTCTACAACAGCGGTGGAAGGCGAATGCGAAATGC |  |  |
| *TcG4* | *T. cruzi* G4 | AY727917.1 | F | GGAAGGGTCGACGCCACCATGTCAGCCAAGGCTCCCCCCAAAACAC | 276 |  |
|  |  |  | R | GGAAGCAGATCTCTACTTTTCAAGCGCCTTAACAAGCTCG |  |  |
| **Tissue levels of parasite DNA** | | | | | |  |
| *Tc18SrDNA* | *T. cruzi* 18S ribosomal DNA | [AJ009147.1](http://www.ncbi.nlm.nih.gov/nucleotide/401424696?from=982402&to=991477&report=gbwithparts) | F | TTTTGGGCAACAGCAGGTCT | 199 |  |
|  |  |  | R | CTGCGCCTACGAGACATTCC |  |  |
| *Gapdh* | GAPDH | NM_001289726.1 | F | AACTTTGGCATTGTGGAAGG | 223 |  |
|  |  |  | R | ACACATTGGGGGTAGGAACA |  |  |
| **Gene expression (mRNA/cDNA levels)** | | | | | |  |
| *Nppa* | ANP | NM_008725.3 | F | GAGGAGAAGAGCCGGTA | 220 |  |
|  |  |  | R | CTAGAGAGGGAGCTAAGTG |  |  |
| *Nppb* | BNP | NM_008726.5 | F | TGATTCTGCTCCTGCTTTTC | 82 |  |
|  |  |  | R | GTGGTTGTTCTGGAGACTG |  |  |
| *Myh7* | MHC-β | XM_017315841.2 | F | CACCAGCTCCGATGCAAT | 84 |  |
|  |  |  | R | AGGATGCGATACCTCTGCCG |  |  |
| *Tagln* | SM22*-α/TAGLN* | U36588.1 | F | GATGGAACAGGTGGCTCA | 161 |  |
|  |  |  | R | TTCCATCGTTTTTGGTCA |  |  |
| *Acta1* | ACTA1  (α-sk Actin) | BC064800.1 | F | CTGACAGAGGCACCACTGAA | 160 |  |
|  |  |  | R | CATCTCCAGAGTCCAGCACA |  |  |
| *Gsk3b* | GSK3B | NM_019827.6 | F | CAGTGGTGTGGATCAGTTGG | 232 |  |
|  |  |  | R | ATGTGCACAAGCTTCCAGTG |  |  |
| *Col1a1* | COL1a1 | NM_007742.4 | F | GAGCGGAGAGTACTGGATCG | 158 |  |
|  |  |  | R | GCTTCTTTTCCTTGGGGTTC |  |  |
| *Col3a1* | COL3a1 | NM_009930.2 | F | GTCCACGAGGTGACAAAGGT | 204 |  |
|  |  |  | R | GATGCCCACTTGTTCCATCT |  |  |
| *Col5a1* | COL5a1 | NM_015734.2 | F | GGTCCCTGACACACCTCAGT | 182 |  |
|  |  |  | R | TGCTCCTCAGGAACCTCTGT |  |  |
| *Mmp2* | MMP2 | NM_008610 | F | GCGACCACAACCAACTACGA | 99 |  |
|  |  |  | R | TGGCATGGCCGAACTCAT |  |  |
| *Mmp3* | MMP3 | [NM_010809.2](https://www.ncbi.nlm.nih.gov/nucleotide/NM_010809.2?report=genbank&log$=nucltop&blast_rank=1&RID=SA5DZA9B01R) | F | CCCCTGATGTCCTCGTGGTA | 99 |  |
|  |  |  | R | AGGGTGCTGACTGCATCAAAG |  |  |
| *Mmp8* | MMP8 | [NM_008611.4](https://www.ncbi.nlm.nih.gov/nucleotide/NM_008611.4?report=genbank&log$=nucltop&blast_rank=1&RID=SA5PXBG3014) | F | CTCGTGGCTGCTCATGAATTT | 70 |  |
|  |  |  | R | ACATCAAGGCACCAGGATCAG |  |  |
| *Mmp9* | MMP9 | NM_013599 | F | CCTACTGCGGGCTCTTCTGA | 70 |  |
|  |  |  | R | CATCCACATTGCAAGGATTGTC |  |  |
| *Mmp12* | MMP12 | NM_008605 | F | GGGCTGCAGCATTCCAATAA | 90 |  |
|  |  |  | R | GTCATCAGCAGAGAGGCGAAA |  |  |
| *Mmp13* | MMP13 | NM_008607 | F | GAAGACCCCAACCCTAAGCAT | 80 |  |
|  |  |  | R | CGGAGACTGGTAATGGCATCA |  |  |
| *Il1b* | IL-1β | NM_008361 | F | CAACCAACAAGTGATATTCTCCATG | 152 |  |
|  |  |  | R | GATCCACACTCTCCAGCTGCA |  |  |
| *Il6* | IL-6 | NM­_031168 | F | GAGGATACCACTCCCAACAGACC | 140 |  |
|  |  |  | R | AAGTGCATCATCGTTGTTCATACA |  |  |
| *Tnfa* | TNF-α | NM_013693 | F | CATCTTCTCAAAATTCGAGTGACAA | 174 |  |
|  |  |  | R | TGGGAGTAGACAAGGTACAACC |  |  |
| *Tgfb1* | TGF-β | NM_011577 | F | CCTGTCCAAACTAAGGC | 207 |  |
|  |  |  | R | GGTTTTCTCATAGATGGC |  |  |
| *Gapdh* | GAPDH | NM_001289726.1 | F | AACTTTGGCATTGTGGAAGG | 223 |  |
|  |  |  | R | ACACATTGGGGGTAGGAACA |  |  |
