# Supplementary material for: Antigen-Based Nano-Immunotherapy Controls Parasite Persistence, Inflammatory and Oxidative Stress, and Cardiac Fibrosis, the Hallmarks of Chronic Chagas Cardiomyopathy, in A Mouse Model of Trypanosoma cruzi Infection
Source: Vaccines (Basel). 2020 Feb 21;8(1):96. doi: 10.3390/vaccines8010096 (PMC7157635; doi:10.3390/vaccines8010096)
Supplement: Supplementary file 1 [file vaccines-08-00096-s001.zip › Supp file 2 Figure S1-S3.docx]

Supplementary file 2: Supplemental figures and figure legends


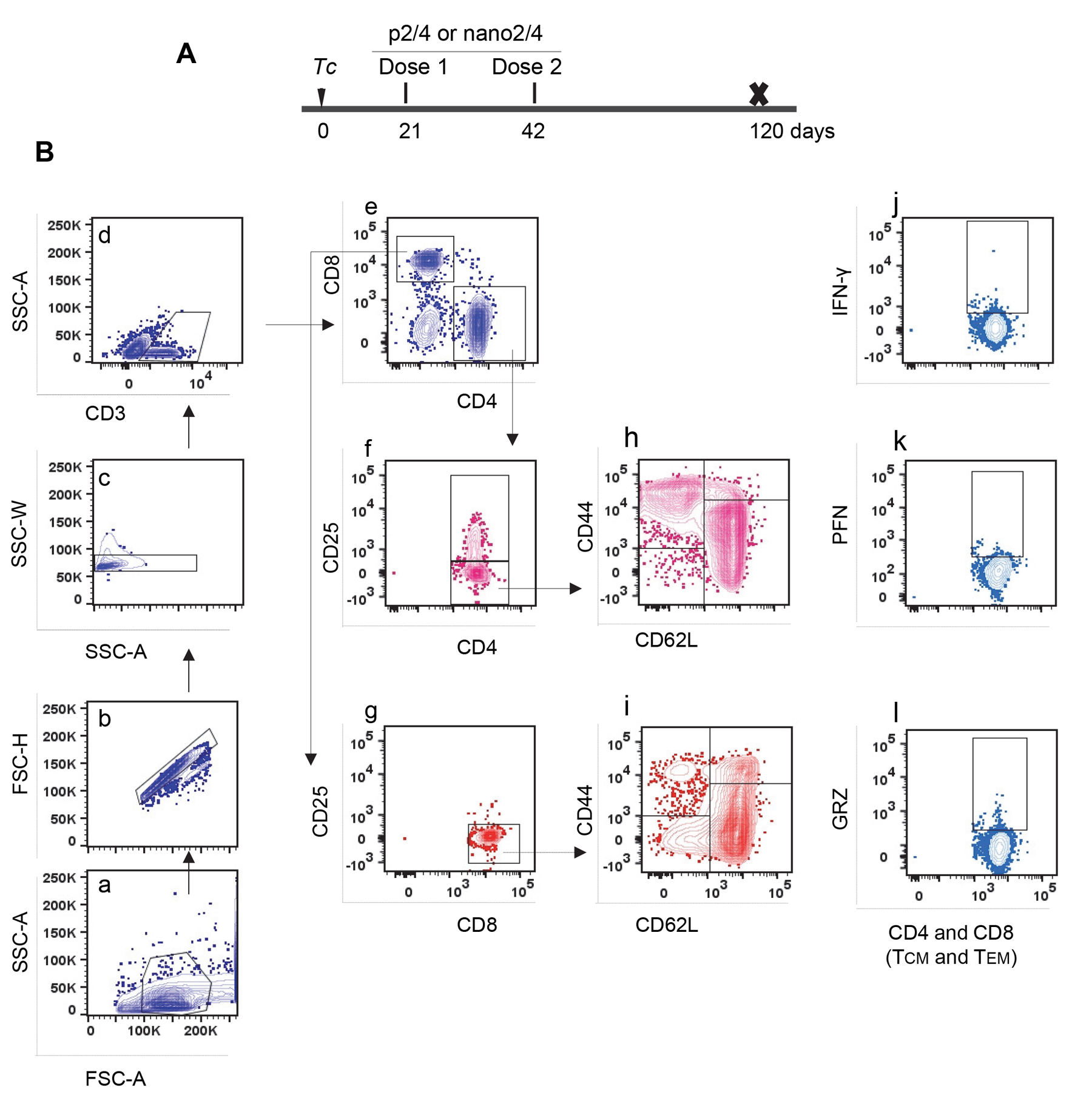


**Figure 1. (A) Schematic of experimental design.** Mice (C57BL/6, females, 6 weeks age) were challenged with *T. cruzi* (SylvioX10, 10,000 trypomastigotes per mouse) and treated with two doses of TcG2 and TcG4 cloned in pCDNA3.1 (referred as p2/4) or nano plasmid (referred as nano2/4) at day 21 day and 42 post-infection. All mice were euthanized at 120 days pi corresponding to chronic phase. **(B) Schematic showing gating strategy for CD4^+^ and CD8^+^ cell phenotypes.** Single cell suspensions of splenocytes were stained with fluorescence-conjugated antibodies to surface and intracellular markers and analyzed by flow cytometry. Live cells were gated in forward and side scatter (a), excluded of doublets in b & c and splenocytes were gated for CD3^+^ cells (d) to evaluate frequency of CD4^+^ and CD8^+^ cells (e). CD4^+^ and CD8^+^ T cells were further gated against CD25 to remove potential T_Reg_ cells (f & g). CD4^+^CD25^-^ and CD8^+^CD25^-^ cells were further screened to distinguish naïve (CD44^lo^CD62L^hi^), central memory (T_CM_, CD^44hi^CD62L^hi^), and effector/effector memory (T_EM_, CD44^hi^CD62L^lo^) phenotypes (h & i). Each sub-population (T_CM_ and T_EM_) of CD4^+^ and CD8^+^ T cells were also analyzed for the expression of granzyme B (GRZ), IFN-γ, and perforin (PERF) (j, k, l).


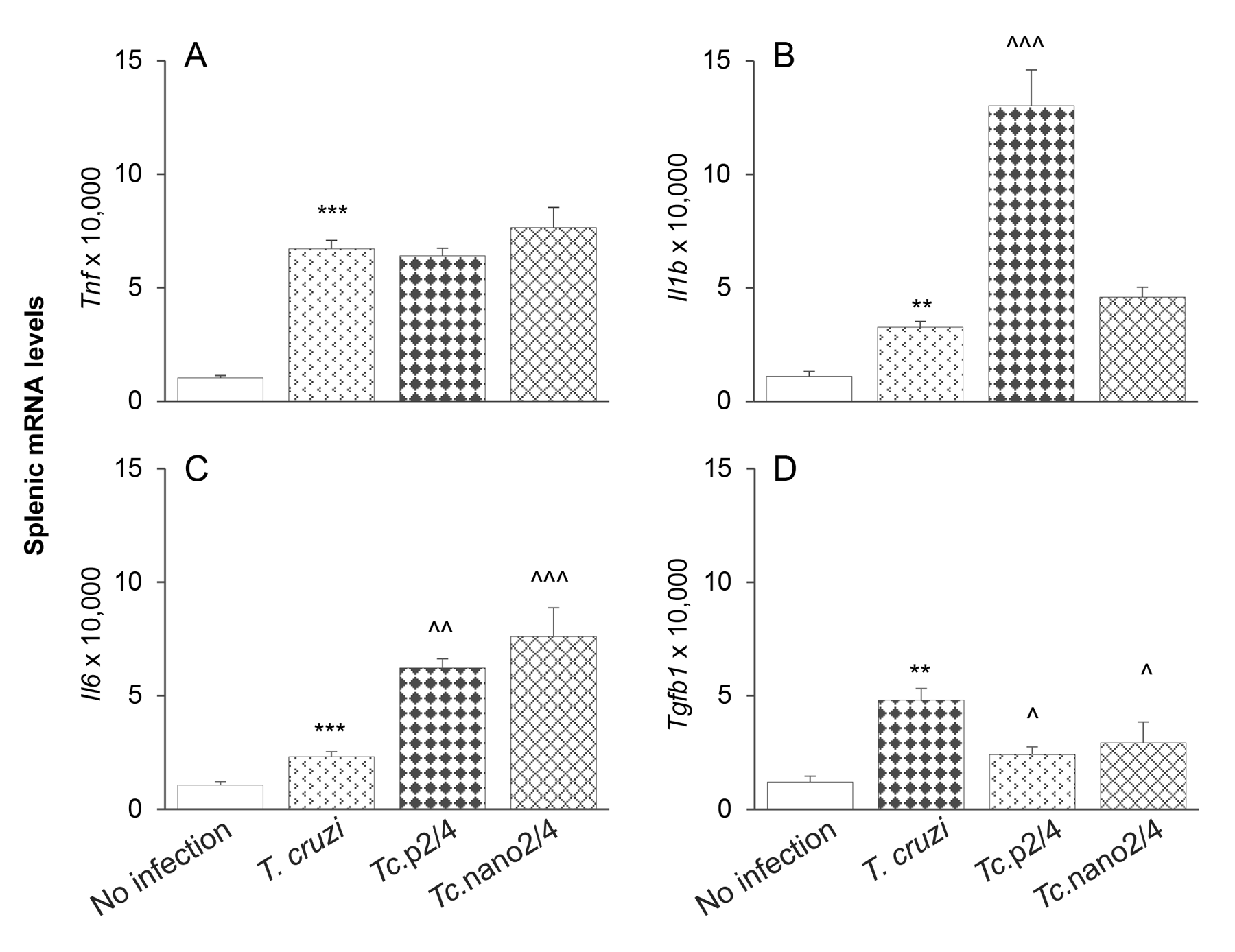


**Figure 2. Splenic expression of cytokines in Chagas mice (± immune therapy).** Mice were challenged, treated, and euthanized as in Figure S1A. Splenocytes were examined by real-time RT-qPCR to detect **(A)** *Tnf*, **(B)** *Il1b* **(C)** *Il6*, and **(D)** *Tgfb1* mRNA levels (normalized to murine Gapdh). Data (mean ± SD) are representative of duplicate observations per sample (n = 5 mice per group). Significance was calculated by student’s t-test (* no infection vs. *Tc*) and 1-way ANOVA/Tukey’s post-hoc test (^ *Tc* vs. *Tc*.p2/4 or *Tc*.nano2/4 and ^&^ *Tc*.p2/4 vs. *Tc*.nano2/4). The p values of <0.05, <0.01, and <0.001 are presented with one, two, and three symbol characters, respectively.


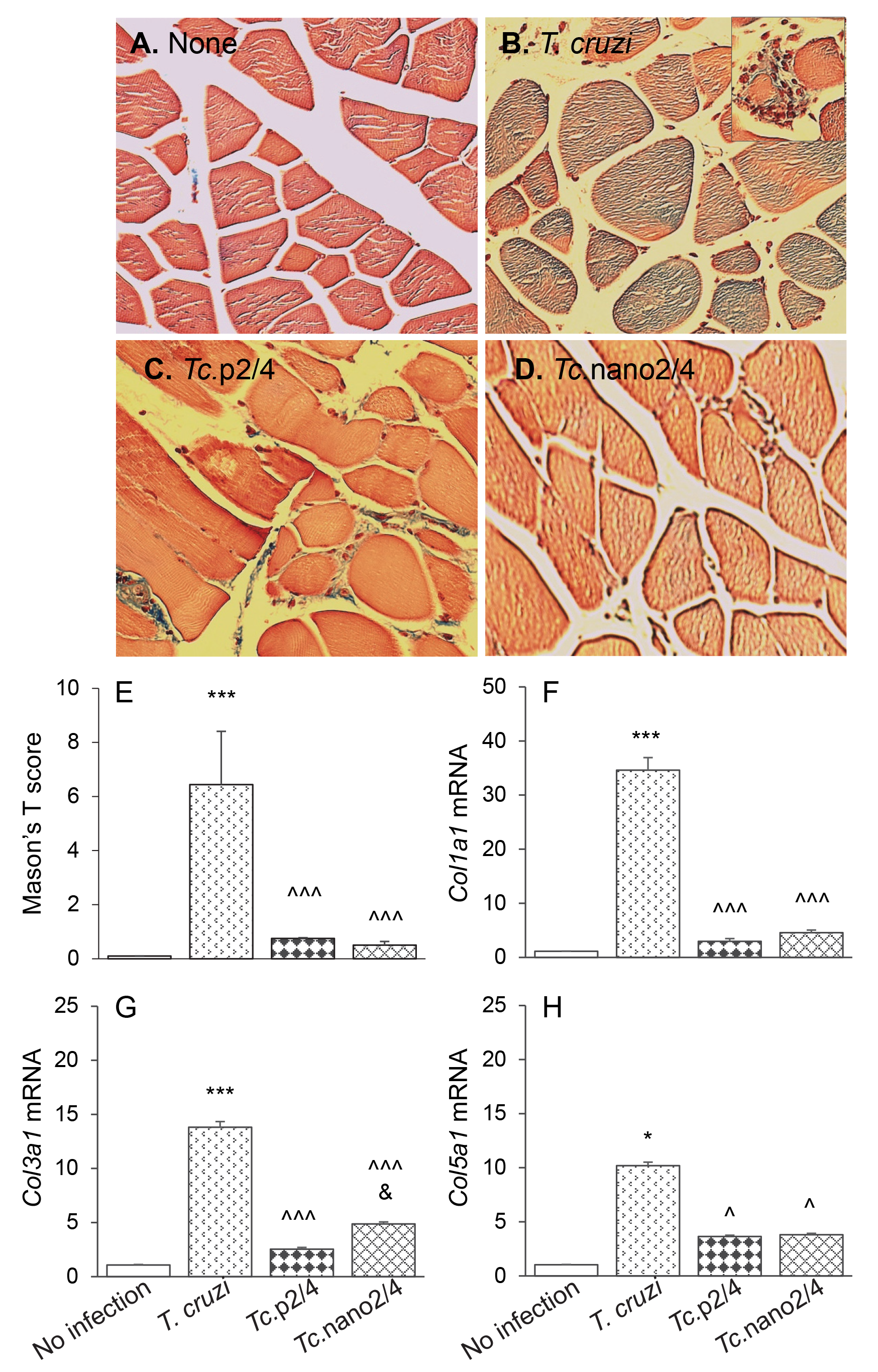


**Figure S3. Skeletal muscle fibrosis in chronically infected mice (± nano immune therapy).** Mice were infected, treated and euthanized as described in S1 Figure. **(A-E)** Paraffin-embedded skeletal muscle sections (5 µm) were examined by Masson’s trichrome staining. Shown are representative images (collagen fibers: blue, nuclei: black, background: red) of tissue sections from non-infected **(A)**, infected/non-treated **(B)**, *Tc.*p2/4 **(C)**, and *Tc.*nano2/4 **(D)** groups of mice during chronic disease phase (150 days post-infection). **(E)** Masson’s T score (mean value ± SD) was calculated as described in Materials and Methods (n=3 mice per group, > two tissue sections per mouse, > 9 microscopic fields per section). **(F-H)** Real time RT-qPCR evaluation of *Col1a1, Col3a1, and Col5a1* mRNA levels. Data were normalized to Gapdh and values are presented as mean ± SD (n = 5 mice per group, duplicate observations per sample). Significance was calculated and annotated as described in S2 Figure.
